# The monitoring of gene functions on a cell-defined siRNA microarray in human bone marrow stromal and U2OS cells

**DOI:** 10.1016/j.dib.2016.03.040

**Published:** 2016-03-12

**Authors:** Hi Chul Kim, Gi-Hwan Kim, David Shum, Ssang-Goo Cho, Eun Ju Lee, Yong-Jun Kwon

**Affiliations:** a Institut Pasteur Korea, IP-Korea, 696 Sampyeong-dong, Bundang-gu, Seongnam-si, Gyeonggi-do 463-400, Republic of Korea; b Biomedical Research Institute & IRICT, Seoul National University Hospital, Seoul, Republic of Korea; c Department of Animal Biotechnology (BK21), Animal Resources Research Center, Konkuk University, Seoul 143-702, Republic of Korea

## Abstract

Here, we developed a cell defined siRNA microarray (CDSM) for human bone marrow stromal cells (hBMSCs) designed to control the culture of cells inside the spot area without reducing the efficiency of siRNA silencing, “Development of a cell-defined siRNA microarray for analysis of gene functionin human bone marrow stromal cells” (Kim et al., 2016 [1]). First, we confirmed that p65 protein inhibition efficiency was maintained when hBMSCs were culture for 7 days on the siRNA spot, and siRNA spot activity remained in spite of long term storage (10 days and 2 months). Additionally, we confirmed p65 protein inhibition in U2OS cells after 48 h reverse transfection.

**Specifications table**

**Table:** 

Subject area	*Biology*
More specific subject area	*siRNA*, *high-throughput screening, cell-based microarray*
Type of data	*Figure*, *graph*
How data was acquired	*Microscopy, point scanning confocal microscope*
Data format	*Analyzed*
Experimental factors	*hBMSCs and U2OS cells were cultured on the array and induced knockdown of p65 by siRNA spot*
Experimental features	*hBMSCs were seeded on siRNA spot array and incubated for 10 min for cell attachment and then unattached cells were removed. Cell-spots were incubated for 2 days and 7 days to induce knock down by siRNA spot. This experiment was performed for confirmation of siRNA spot activity by reverse transfection time and by storage period of siRNA spot array*. *U2OS cells were carried out by siRNA reverse transfection for 2 days following with same process as hBMSCs*
Data source location	*Institut Pasteur Korea*, *IP-Korea*, *Seongnam-si*, *Republic of Korea*
Data accessibility	*Data is provided within the article*

**Value of the data**

• This data shows that CDSM is a useful tool for image-based siRNA screening with cellular models.
• This data shows that the preservation of siRNA stability in CDSM offers opportunity for siRNA screening.
• This data proves that CDSM technology can be applied to hBMSCs and U2OS cells to identify gene functions.

## 1 Data

**Fig. 1:**
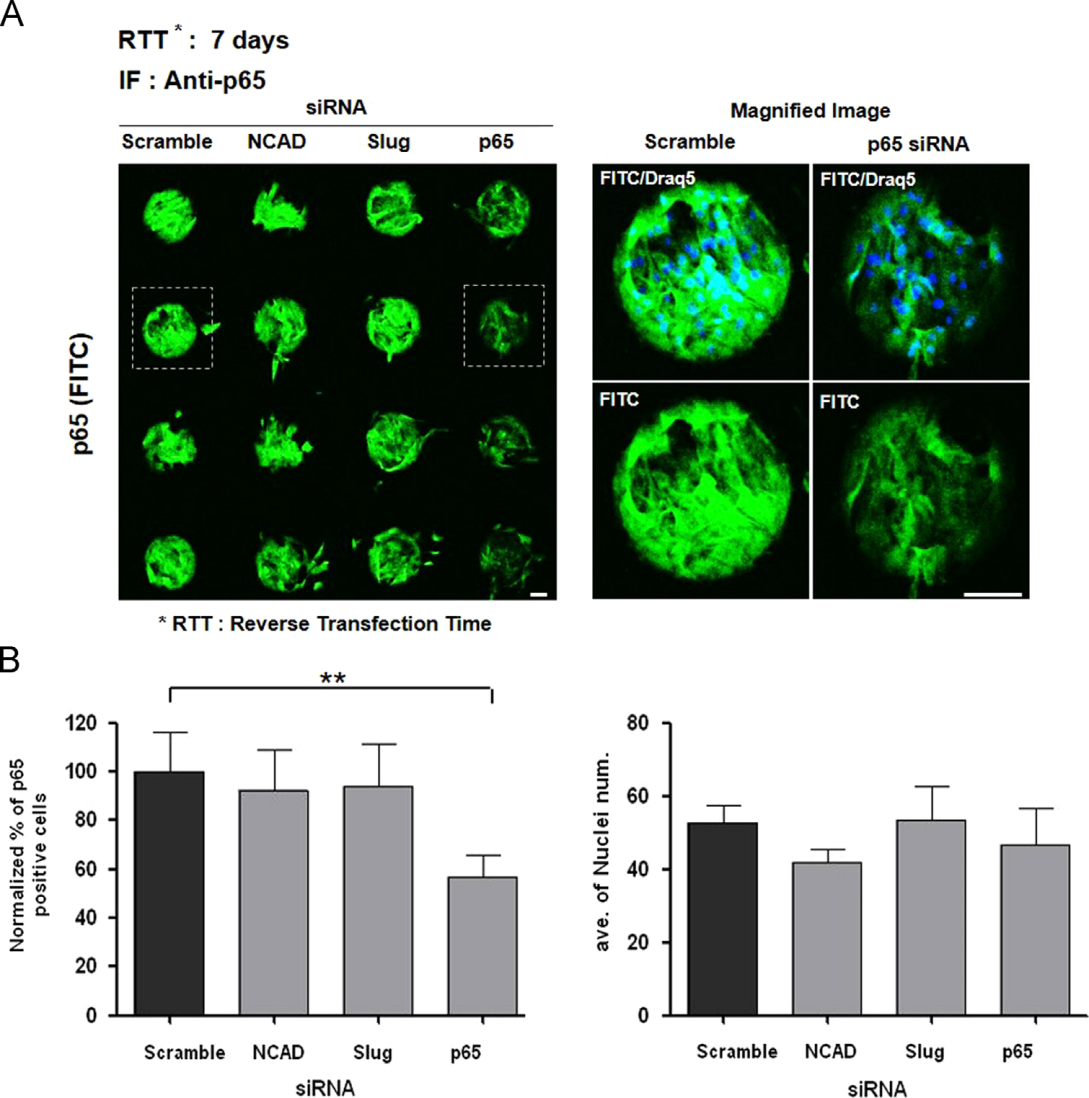
(A) Immunofluorescence image of p65 siRNA knock down effect after reverse transfection for 7 days (*n*=6 array spots) in hBMSCs. (B) Quantitative analysis for p65 positive cells ratio and cell numbers of (A). The figure images were acquired by an ImageXpress Ultra point scanning confocal microscope with an anti-p65 antibody. Blue: Draq5 for the nucleus, Green: fluorescence-labeled antibody to p65 protein. Scramble siRNA was used control. Each error bar represents the mean±SD. (**p<0.01: statistical significance comparison of scramble siRNA). Scale bar=100 µm.

**Fig. 2:**
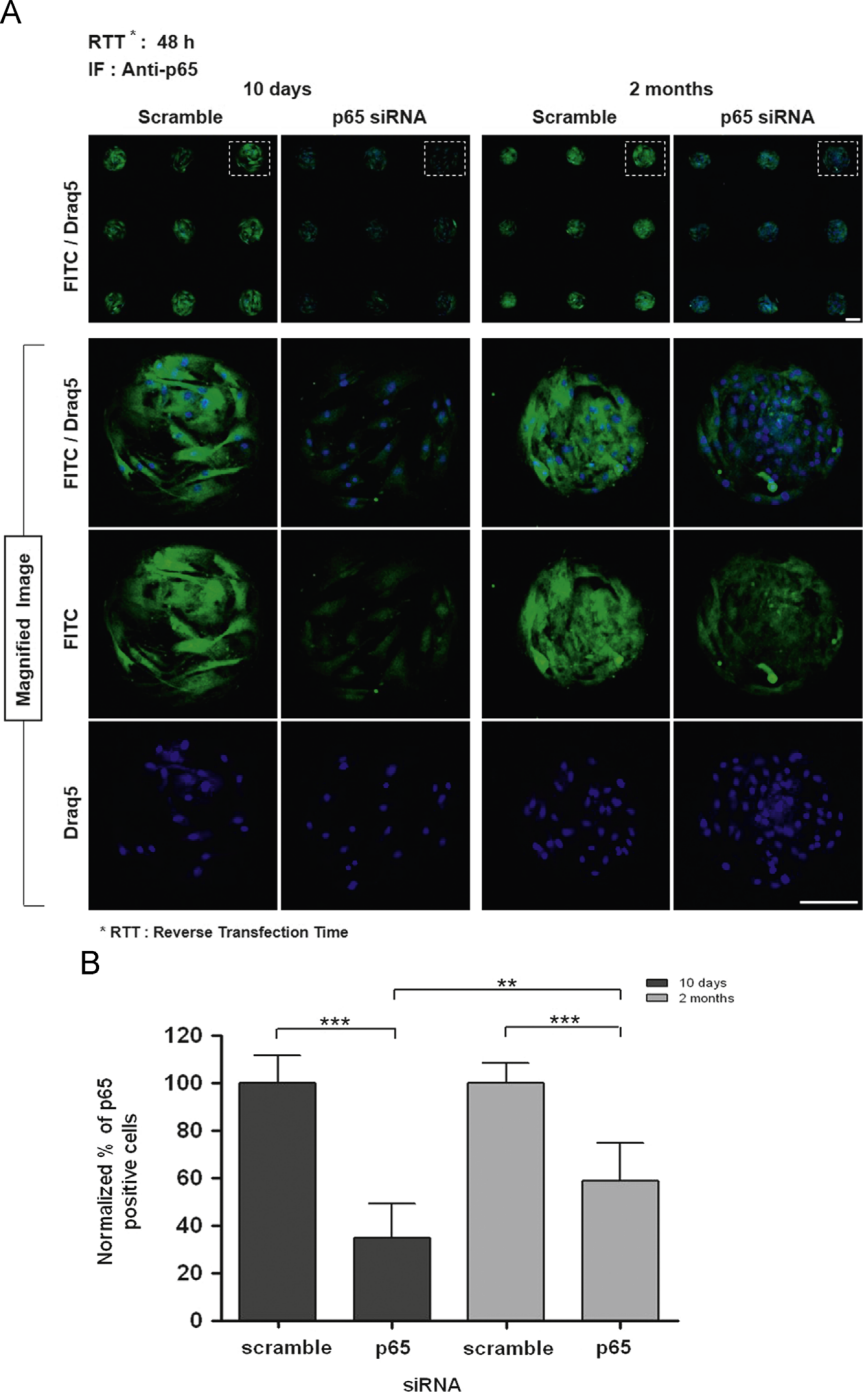
The examination of siRNA activity from cell-defined microarray by storage period in hBMSCs. (A) The immunofluorescence image of p65 siRNA knock down effect after cell defined siRNA microarray stored for 10 days and 2 months at 4 °C (*n*=9 array spots, scale bar=100 µm). (B) Quantitative analysis for p65 positive cells ratio of (A). The figure images were acquired by an ImageXpress Ultra point scanning confocal microscope with an anti-p65 antibody. Blue: Draq5 for the nucleus, Green: fluorescence-labeled antibody to p65 protein. Scramble siRNA was used control. Each error bar represents the mean±SD. (***p<0.001: statistical significance comparison of scramble siRNA, **p<0.01: statistical significance in comparison between the p65siRNA condition of 10 days and 2 months).

**Fig. 3:**
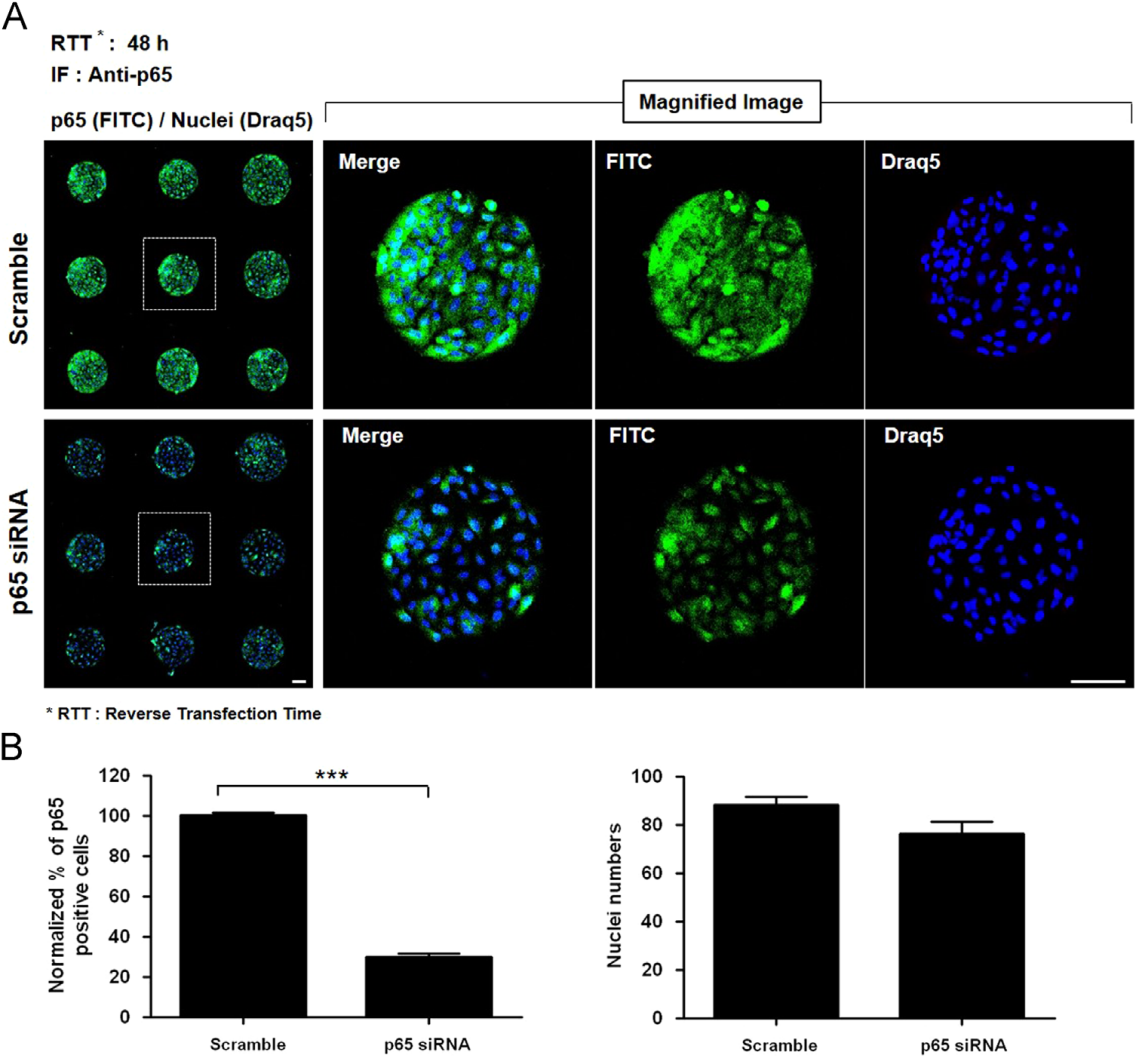
Confirmation of the silencing effects of siRNA by the CDSM in U2OS cells. (A) Immunofluorescence image of p65 silencing by the CDSM. (B) Quantitative analysis of p65-positive cell ratios and nucleus numbers from (A) (*n*=9 array spots, scale bar=100 µm). The figure images were acquired by an ImageXpress Ultra point scanning confocal microscope with an anti-p65 antibody. Blue: Draq5 for detection of nuclei, green: fluorescence-labeled antibody targeting p65 protein. Scramble siRNA was used as a control. Bars and error bars represent means±SDs. ***p<0.001: statistical significance in comparison with scramble siRNA. Scale bar=100 µm.

We confirmed p65 gene expression knock down persistence effect for 7 days on CDSM (ave. 43.3%) (Fig. 1). We identified p65 protein inhibition effect, 65.4% (10 days) and 41.3% (2 months) (Fig. 2). We verified significant efficiency on p65 inhibition from CDSM in U2OS (Fig. 3).

## 2 Experimental design, materials and methods

All experimental design, materials and methods were based on reported paper [1]

### 2.1 hBMSCs and U2OS cells culture

hBMSCs, human bone marrow stromal cells, (Lonza, Basel, Switzerland) were cultured in MSC basal medium (MSCBM; Lonza), supplemented with BMSC growth medium (MSCGM), an hBMSCs SingleQuots kit (Lonza), 5% fetal bovine serum, 1% l-glutamine, and 0.1% GA-1000. U2OS cell cultures were maintained at 37 °C in a humidified cell incubator with an atmosphere of 5% CO_2_, and the culture medium was changed twice per week.

U2OS osteosarcoma cells, (ATCC, Manassas, VA, USA) were cultured in DMEM high-glucose medium supplemented with 10% fetal bovine serum (FBS) and 1% penicillin/streptomycin in a humidified atmosphere of 5% CO_2_at 37 °C.

### 2.2 Composition of the siRNA solution

3 µL of 20 µM siRNA, 3.75 µM as the final concentration, (scramble, p65, Slug, NCAD siRNA as a smart pool of four individual siRNAs per target; Dharmacon, Lafayette, CO, USA) was transferred to the 384-well V-bottom plate (Greiner Bio-one, Monroe, NC, USA) containing 2 µL of dH_2_O and 2 µL of 0.6 M sucrose, 80 mM as the final concentration, (Invitrogen, Carlsbad, CA, USA) diluted in Opti-MEM (Gibco, Grand Island, NY, USA). 2 µL of RNAi-Max [Invitrogen] was added and then the solution was mixed gently. The complexes were incubated for 20 min at room temperature after centrifugation (1200 rpm, 30 s). 5 µL of 0.4% gelatin (diluted in dH_2_O, 0.13% final concentration, Invitrogen) and 2 µL of growth factor reduced phenol red-free matrigel (BD Biosciences, San Jose, CA, USA) were added and centrifuged (1200 rpm, 30 s) after mixing gently by pipetting.

### 2.3 Printing for CDSM

Three-dimensional (3D) hydrogel-coated slides (H; 25×75.6×1 mm^3^; SCHOTT, Elmsford, NY, USA) were held at room temperature for 30 min to 1 h before opening the packaging bag. The prepared siRNA transfection solution was printed on the 3D hydrogel-coated slide using SMP9 stealth pins (Telechem, Atlanta, GA, USA) in a high-throughput microarray printer (Genomic Solutions, Ann Arbor, MI, USA) at 22–25 °C and 55–60% relative humidity. Printed spots were 250–350 µm in diameter with a 700 µm spot-to-spot interval. The prepared CDSMs were dried in a desiccating chamber overnight, packaged in airtight bags, and then stored at 4 °C until further use.

### 2.4 hBMSCs and U2OS preparation, culture on the CDSMs

hBMSCs and U2OS cells stored in liquid nitrogen (LN_2_) tank were thawed into T25 or T75 flasks and then cultured for 1 week to reach 70–80% confluence. The prepared cells (2×10^5^ cells/4 mL/slide) were detached with 0.05% trypsin-EDTA (Gibco) then seeded onto CDSMs which were removed from moisture and transferred to a 4-well rectangular dish (Nunc, Rochester, NY, USA). Cell attachment was induced for 10 min, and unattached cells were removed by washing 3–5 times with fresh hBMSCs and DMEM medium. The attached cells on the spot region were cultured 2 days and 7 days for reverse transfection.

### 2.5 Immunofluorescence staining

The immunofluorescence staining of hBMSCs and U2OS cells on CDSMs was performed using standardized methods. The cells were washed with 1×phosphate-buffered saline (PBS) once and fixed with 4% (w/v) paraformaldehyde in 1×PBS for 10 min. Cells were then washed again with 1×PBS and permeabilized with 0.001% Triton-X100 in 1×PBS for 10 min. Primary antibody targeting p65 (1:400 dilution; rabbit polyclonal; Santa Cruz Biotechnology, Dallas, TX, USA) were diluted with 10% goat serum in 1×PBS and incubated for 1.5 h at room temperature. After washing three times with 1×PBS, secondary goat anti-rabbit conjugated with Alexa 488 were diluted with 10% goat serum in 1×PBS and incubated for 1.5 h at room temperature. After washing three times with 1×PBS, 5 mM DRAQ5 (1:2000 in PBS; Biostatus, Leicestershire, UK) was added, and samples were incubated for 10 min to stain the nuclei. After rinsing with 1×PBS, 2–3 drops of mounting solution (Dako, France) was added to the CDSM, and the slides were covered with glass coverslips (24×60 mm^2^; Marienfeld, Germany), ensuring that there were no air bubbles. Slides were then dried for 1 h at room temperature. After fixing with mounting solution, the CDSMs were stored at 4 °C until image acquisition.

### 2.6 Image acquisition and analysis

Immunofluorescence imaging was performed using an ImageXpress Ultra point scanning confocal microscope (Molecular Devices, Sunnyvale, CA, USA) equipped with four solid-state lasers for simultaneous excitation at 405, 488, 561, and 635 nm; a galvanometer for X scanning; and a stage for Y scanning. Each cell spot was scanned at 10×or 20×magnification using a Nikon air immersion objective lens with specific filter sets for Alexa488. The acquired images were analyzed with MetaXpress software (Molecular Devices). Cell scoring parameters were also determined to evaluate the proportion of fluorescent-positive cells which was calculated according to the fluorescence intensity, nuclei numbers, and size. GraphPad Prism 5 software (GraphPad Software, La Jolla, CA, USA) was used to determine statistical significance for comparisons of two groups by one-way or two-way analysis of variance (ANOVA) and the Bonferroni method. Differences with *p* values of less than 0.05 were considered statistically significant.
