# Supplementary material for: The monitoring of gene functions on a cell-defined siRNA microarray in human bone marrow stromal and U2OS cells
Source: Data Brief. 2016 Mar 12;7:673–8. doi: 10.1016/j.dib.2016.03.040 (PMC4802669; doi:10.1016/j.dib.2016.03.040)
Supplement: Supplementary file 1 — Supplementary material [file mmc1.docx]

*Data article*

**Title:** The monitoring of gene functions on a cell-defined siRNA microarray in human bone marrow stromal and U2OS cells

**Authors:** Hi Chul Kim^a,c^, Gi-Hwan Kim^b^, David Shum ^a^, Ssang-Goo Cho^c^, Eun Ju Lee^b,^*, Yong-Jun Kwon^a,d,^*

**Conflict of interest: none**
